# Phonon laser in a cavity magnomechanical system

**DOI:** 10.1038/s41598-019-52050-7

**Published:** 2019-10-31

**Authors:** Ming-Song Ding, Li Zheng, Chong Li

**Affiliations:** 1 School of Physics, Dalian University of Technology, Dalian, 116024 China; 2 Science and Engineering College, Dalian Polytechnic University, Dalian, 116034 China

**Keywords:** Quantum physics, Optical physics

## Abstract

Using phonons to simulate an optical two-level laser action has been the focus of research. We theoretically study phonon laser in a cavity magnomechanical system, which consist of a microwave cavity, a sphere of magnetic material and a uniform external bias magnetic field. This system can realize the phonon-magnon coupling and the cavity photon-magnon coupling via magnetostrictive interaction and magnetic dipole interaction respectively, the magnons are driven directly by a strong microwave field simultaneously. Frist, the intensity of driving magnetic field which can reach the threshold condition of phonon laser is given. Then, we demonstrate that the adjustable external magnetic field can be used as a good control method to the phonon laser. Compared with phonon laser in optomechanical systems, our scheme brings a new degree of freedom of manipulation. Finally, with the experimentally feasible parameters, threshold power in our scheme is close to the case of optomechanical systems. Our study may inspire the field of magnetically controlled phonon lasers.

## Introduction

In recent years, the cavity magnomechanical systems has been becoming a novel platform for realizing the coupling between photons, magnons and phonons. Among them, the coupling between photons and magnons is realized by the magnetic dipole interaction, and magnetostrictive force is a key to the magnon-phonon coupling. As we know, traditional optomechanical systems utilize radiation force^1–17^, electrostatic force^18,19^, and piezoelectric force^20^ for the interaction between phonons and cavity photons. However, the adjustability of them are not very good. The emergence of magnetostrictive force brings us a new way to achieve different information carriers^21,22^. And a small yttrium iron garnet (YIG) sphere is introduced into the cavity magnomechanical system as an effective mechanical resonator. The varying magnetization caused by the excitation of the magnons in the YIG sphere results in the geometric deformation of the surface, and it leads to the magnon-phonon coupling. Here, the magnons inside the YIG sphere can be considered as collectively excited, and the frequency of them can be controlled by an external magnetic field.

Because the YIG sphere has characteristics of high density and low loss, the Kittel mode^23^ (a ferromagnetic resonance mode) in it can be strongly coupled^24–26^ with the cavity mode. In addition, the YIG sphere has nonlinearity and adjustability in many quantum information carriers, these excellent properties make it possible to find many interesting and important phenomena in cavity-magnon systems and cavity magnomechanical systems. Based on it, a lot of theoretical and experimental researches have been done. J. Q. You *et al*. have found the bistability of cavity magnon polaritons^27^, G. S. Agarwal *et al*. have discussed the tripartite entanglement among magnons, cavity photons, and phonons^21^. Furthermore, high-order sideband generation^28,29^, magnon Kerr effect^30^, the light transmission in cavity-magnon system^31^ and other researches were also studied^32–37^.

Phonon laser as a novel laser has been developed rapidly, it generates coherent sound oscillations (mechanical vibration) by optical pumping. The phonon laser^38–40^can be considered as an analogue of a two-level optical laser^41,42^. We use two optical supermodes to correspond to the ground and excited states, respectively. Between the two supermodes, phonons serve as the medium of transition. As early as 2003, Chen. J and Khurgin have proved the feasibility of phonon laser and given an effective scheme^43^. Then people began to use trapped ions and quantum dots to realize phonon laser^44–46^. Up to now, numerous theoretical and experimental researches have been proposed, such like a cavity optomechanics-based low threshold phonon laser^38^, a phonon laser enhanced by ${\mathscr{PT}}$-symmetric^39^, the nonreciprocal phonon lasering in a cavity optomechanical system^40^, the influence of exceptional point on phonon lasers^47^, the scheme of amplifying phonon laser by phonon stimulated emission coherence^48^, the phonon-stimulated emission in cryogenic ionic compounds^49,50^, semiconductor superlattices^51^ and so on^48,52–55^. In addition, the phonon laser have also attracted extensive interest in medical imaging and high-precision measurement equipment.

In this work, we study a cavity magnomechanical system, where a YIG sphere is placed in a microwave cavity, there is an uniform external bias magnetic field $H$ in the vertical direction simultaneously. The magnetostrictive (radiation pressure like) leads to the magnon-phonon coupling, and the photons and magnons are coupled via magnetic dipole interaction. It is worth noting that unlike optical pump in the traditional cavity optomechanical system, we introduce a magnetic drive field to to realize phonon laser. Furthermore, the magnomechanical interaction which is quite weak in experiments can be enhanced by the gain of magnon mode. We found that the adjusted applied magnetic field $H$ can be regarded as a good control method to the phonon laser. Compared with phonon laser in optomechanical systems, our scheme brings an additional degree of freedom of manipulation. The threshold conditions of drive magnetic field intensity for phonon laser is obtained. We can make our system reach the threshold condition by enhancing drive magnetic field. And the threshold power required can be below $10\ \mu$W within the experimental allowable range of parameters. According to recent work, the threshold power in cavity optomechanical system is generally about $7\ \mu$W^38–40^.

## Results

### Model and dynamical equations

**Fig. 1:**
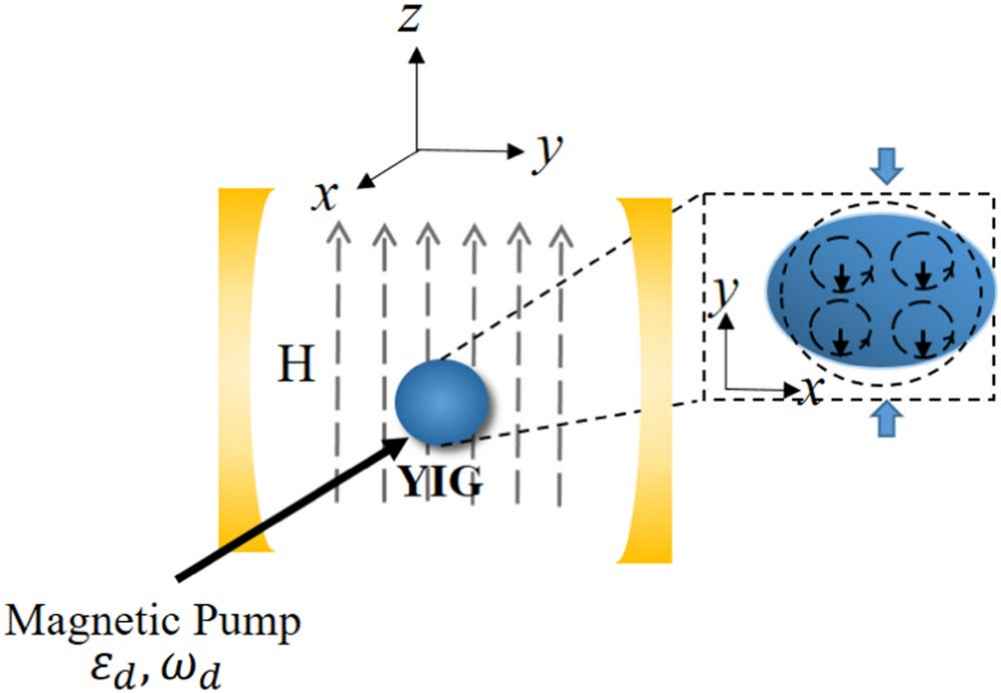
Schematic diagram of our system. The YIG sphere is placed in the maximum magnetic field of the microwave cavity mode. Applying an external magnetic field $H$ along z-direction makes YIG sphere produce uniform magnon mode. The enlarged YIG sphere on the right shows the magnetization of magnon (black down arrows), which leds to the micro-deformations on the surface of spheres (y-direction). Conversely, the deformation can also lead to changes in magnetization of magnetons.

In Fig. 1, a highly polished single-crystal YIG sphere (a sphere of $1mm$ in diameter is considered in^30^) is placed in a microwave cavity. At the same time, an uniform external bias magnetic field $H$ is introduced in the vertical direction, which establish the magnon-photon coupling^27,30^, and the rate of coupling can be tuned by the position of the sphere. There are three modes in this system: cavity photon mode, magnon mode and phonon mode. In addition, the external bias magnetic field $H$ is created by a high precision tunable electromagnet, and the adjusting range of bias magnetic field $H$ is between $0$ and $1T$^30^.

Here, the coupling between magnons and phonons is generated by magnetostrictive interaction (the derivation of the relevant Hamiltonian can be found in^22^). The magnon excitation causes magnetization to change, which leads to micro deformation of YIG sphere slightly. Based on it, we can consider the sphere as an excellent mechanical resonator, so the phonon mode of the sphere can be introduced. Here, a microwave source is utilized to directly drive the magnon mode and it can enhance the magnomechanical coupling^21,30^. It is worth mentioning that the directions of the applied magnetic field $H$, the drive magnetic field, and the magnetic field of the cavity mode are perpendicular to each other. So we can adjust only one of them without worrying about the impact on the rest. Furthermore, we assume that the size of YIG sphere is much smaller than the wavelength of the cavity. Accordingly, the interaction between cavity photons and phonons can be neglected. The total Hamiltonian of our system reads ($\hslash =1$)

1 $$\begin{array}{ccl}{H}_{total} & = & {H}_{0}+{H}_{int}+{H}_{d},\\ {H}_{0} & = & {\omega }_{a}{a}^{\dagger }a+{\omega }_{m}{m}^{\dagger }m+{\omega }_{b}{b}^{\dagger }b,\\ {H}_{int} & = & {g}_{ma}({a}^{\dagger }m+{m}^{\dagger }a)-{g}_{mb}{m}^{\dagger }m(b+{b}^{\dagger }),\\ {H}_{d} & = & i({\varepsilon }_{d}{m}^{\dagger }{e}^{-i{\omega }_{d}t}-{\varepsilon }_{d}^{* }m{e}^{i{\omega }_{d}t}),\end{array}$$

where ${H}_{0}$ is the free Hamiltonian, ${\omega }_{a}{a}^{\dagger }a$ and ${\omega }_{m}{m}^{\dagger }m$ denote the cavity photon mode and magnon mode, respectively. The third term describes the mechanical mode. ${\omega }_{a}$, ${\omega }_{m}$ and ${\omega }_{b}$ denote the resonance frequencies of the cavity, magnon, and mechanical modes. The frequency of uniform magnon mode in the YIG sphere is ${\omega }_{m}={\gamma }_{g}H$ (${\gamma }_{g}/2\pi =28GHz/T$ is the gyromagnetic ratio). The annihilation (creation) operators of these modes are $a({a}^{\dagger })$, $m({m}^{\dagger })$ and ${b}^{\dagger }(b)$, respectively.

${H}_{int}$ is the interaction Hamiltonian of our system, which consists of the Hamiltonian of photon-magnon coupling and the phonon-magnon interaction. ${g}_{ma}$ and ${g}_{mb}$ are the coupling rates of the magnon-photon interaction and the magnon-phonon interaction, respectively. And ${g}_{ma}$ can be tuned by external magnetic field $H$ or the position of the YIG sphere inside the cavity. Finally, ${H}_{d}$ is the driving field of magnon mode, as shown in^30^, J. Q. You *et al* designed an experimental setup, the YIG sphere can be directly driven by a superconducting microwave line which is connected to the external port of the cavity. Under the case of the low-lying excitations, we have ${\varepsilon }_{d}=\frac{\sqrt{5}}{4}{\gamma }_{g}\sqrt{M}{B}_{0}$, where ${B}_{0}$ and ${\omega }_{d}$ stand for the amplitude and frequency of drive magnetic field^21^, respectively and $M\ =\ \rho V$ is the total number of spins, $V$ is the volume of YIG sphere. Furthermore, $\rho \ =\ 4.22\times 1{0}^{27}{m}^{-3}$ is the spin density of the YIG sphere.

By making a frame rotating at the frequency ${\omega }_{d}$, the total Hamiltonian of the system (under a rotating-wave approximation) is given as

2 $$\begin{array}{lll}{H}_{total} & = & -{\Delta }_{a}{a}^{\dagger }a-{\Delta }_{m}{m}^{\dagger }m+{\omega }_{b}{b}^{\dagger }b+\\  &  & {g}_{ma}({a}^{\dagger }m+{m}^{\dagger }a)-{g}_{mb}{m}^{\dagger }m(b+{b}^{\dagger })\\  &  & +i({\varepsilon }_{d}{m}^{\dagger }-{\varepsilon }_{d}^{* }m),\end{array}$$

where ${\Delta }_{a}\ =\ {\omega }_{d}-{\omega }_{a}$, ${\Delta }_{m}\ =\ {\omega }_{d}-{\omega }_{m}$. The Heisenberg-Langevin equations of the system are written as

3 $$\begin{array}{lll}\dot{a} & = & (i{\Delta }_{a}-{\kappa }_{a})a-i{g}_{ma}m-\sqrt{2{\kappa }_{a}}{a}_{int},\\ \dot{m} & = & (i{\Delta }_{m}-{\kappa }_{m})m-i{g}_{ma}a+i{g}_{mb}m(b+{b}^{\dagger })\\  &  & +{\varepsilon }_{d}-\sqrt{2{\kappa }_{m}}{m}_{int},\\ \dot{b} & = & (-i{\omega }_{b}-{\gamma }_{b})b+i{g}_{mb}{m}^{\dagger }m-{\xi }_{no},\end{array}$$

where ${\gamma }_{b}$ is the loss of mechanical mode, ${a}_{int},{m}_{int}$ and ${\xi }_{no}$ are input noise operators of cavity, magnon and mechanical modes, respectively. ${\kappa }_{m}$ and ${\kappa }_{a}$ are the decay rates of magnon and microwave cavity modes. Like other work^38,40^, under the strong driven of the magnon mode, we have $\left|\left\langle m\right\rangle \right|\ \gg \ 1$ at the steady-state. The cavity-magnon beam splitter interaction can lead to the large amplitude of cavity field, i.e., $\left|\left\langle a\right\rangle \right|\ \gg \ 1$. So we can safely ignore the quantum noise terms if the mean-number behaviors are only interested. Here, the semi-classical Langevin equations of motion are valid. In other words, we can rewrite all operators as their respective expectation values. Then making the left-hand side equal to $0$, the steady-state mean values of the system read

4 $$\begin{array}{lll}{a}_{s} & = & \frac{{g}_{ma}\cdot {m}_{s}}{{\Delta }_{a}-i{\kappa }_{a}},\\ {m}_{s} & = & \frac{{\varepsilon }_{d}}{({\kappa }_{m}-\frac{{g}_{ma}^{2}{\Delta }_{a}}{{\Delta }_{a}^{2}-{\kappa }_{a}^{2}})-i[{\Delta }_{m}+{g}_{mb}({b}_{s}+{b}_{s}^{* })+\frac{{g}_{ma}^{2}{\kappa }_{a}}{{\Delta }_{a}^{2}-{\kappa }_{a}^{2}}]},\\ {b}_{s} & = & \frac{{g}_{mb}{\left|{m}_{s}\right|}^{2}}{{\omega }_{b}-i{\gamma }_{b}}.\end{array}$$

According to the feasible experimental parameters (${g}_{mb} < 1Hz$), ${g}_{mb}({b}_{s}+{b}_{s}^{* })\ \ll \ {\Delta }_{m}$. Under this condition, we approximately have ${\Delta }_{m}+{g}_{mb}({b}_{s}+{b}_{s}^{* }) \sim {\Delta }_{m}$.

### The distribution of steady-state magnon number

**Fig. 2:**
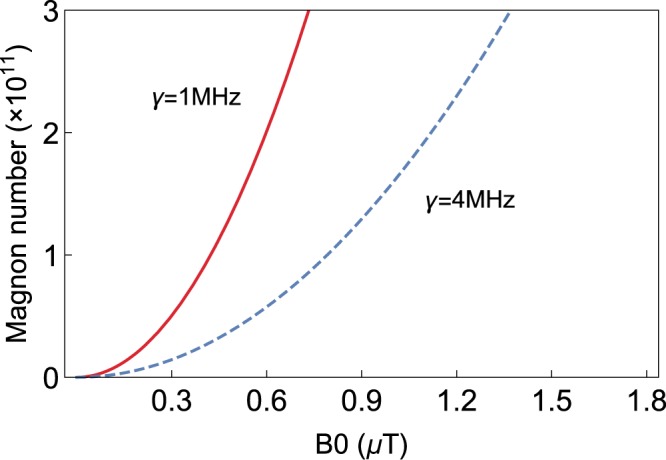
The distribution of steady-state magnon number ${\left|{m}_{s}\right|}^{2}$ versus the drive magnetic field ${B}_{0}$ under $\gamma =1\ MHz$ (red solid line) and $\gamma =4\ MHz$ (blue dashed line). The parameter we used is ${\Delta }_{m}/2\pi =8\ MHz$.

Here, we give the specific values of the parameters used in this paper^22^. ${\omega }_{a}/2\pi ={\omega }_{m}/2\pi =10.1GHz$, ${\omega }_{b}/2\pi =12\ MHz$, ${g}_{ma}/2\pi =6\ MHz$, ${g}_{mb}/2\pi =0.1Hz$, ${\Delta }_{a}/2\pi =8\ MHz$, and the loss of mechanical modes ${\gamma }_{b}/2\pi =100\ Hz$. Our research is in the resolved sideband regime (${\kappa }_{m}/{\omega }_{b} < 1$). The drive power $P=({B}_{0}^{2}/2{\mu }_{0})Ac$^21^, where ${B}_{0}^{2}/2{\mu }_{0}$ is time average of energy per unit volume, $c$ is the velocity of an electromagnetic wave in vacuum, $A$ is the maximum cross-sectional area of YIG sphere. Fig. 2 shows a plot of the steady-state magnon number ${\left|{m}_{s}\right|}^{2}$ versus the drive magnetic field ${B}_{0}$. The number of magnons increases exponentially with the increase of ${B}_{0}$, which represents significant nonlinearity. The corresponding ${B}_{0}$ is much weak relative to the external magnetic field $H$. Furthermore, the results under different losses of supermode $\gamma$ are also given. It can be seen that the smaller the dissipation, the faster ${\left|{m}_{s}\right|}^{2}$ increases.

### Magnetic field-based control of phonon laser action

**Fig. 3:**
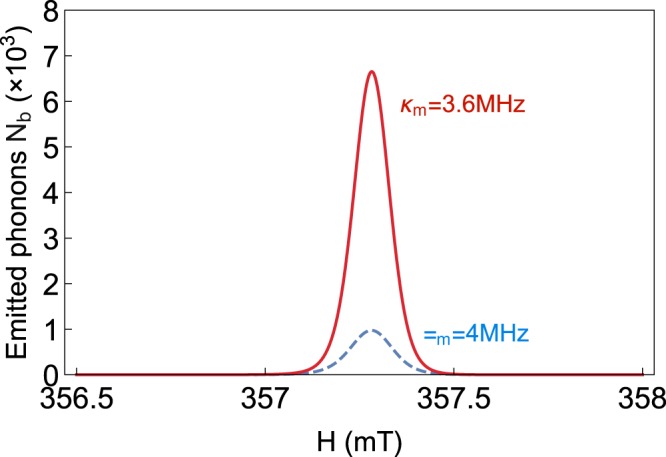
The stimulated emitted phonon number ${n}_{b}$ versus the external bias magnetic field $H$ under ${\kappa }_{m}=3.6\ MHz$ (red solid line) and ${\kappa }_{m}=4\ MHz$ (blue dashed line). The parameter we used is ${\varepsilon }_{d}=5.24\times 1{0}^{12}Hz$ (${B}_{0}=1.8\ \mu T,P=19\ \mu W$) and ${\kappa }_{a}=2.6\ MHz$.

**Fig. 4:**
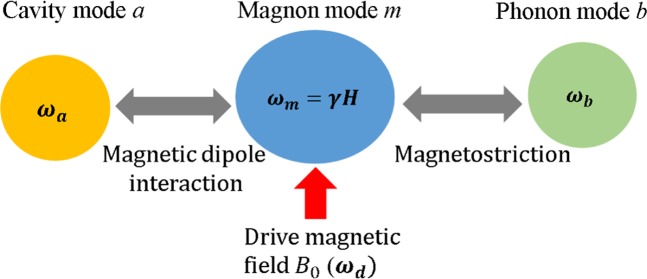
The equivalent coupling model. The frequency of magnon mode is controlled by an external magnetic field $H$, the intensity of the drive magnetic field is also controlled by $b$. The frequency of drive magnetic field is ${\omega }_{d}$.

Here, we study the influence of a magnetic field (including the external bias magnetic field $H$ and the drive magnetic field ${B}_{0}$) on the phonon laser. Figure 3 shows a plot of the phonon number ${n}_{b}$ versus the $H$. There is an obvious window between $H\approx 357\ mT-357.5\ mT$, and a large number of the stimulated emitted phonons appear in this range, this behavior is consistent with previous effect on the number of magnons. The reason for it is when the intensity of the external bias magnetic field $H$ is in the range of $357\ mT-357.5\ mT$, the frequencies of the magnon mode and drive magnetic field can almost reache the condition of resonance (${\omega }_{m}\simeq {\omega }_{d}$). Therefore, the influence of drive magnetic field is the strongest, and the magnetostrictive effect is strengthened effectively^21^, the vibration of the YIG sphere is enhanced due to the magnetostrictive effect simultaneously, which eventually leads to the increase of emitted phonons. Figure 4 shows an equivalent coupling model, it illustrates the coupling between three modes and the effects of magnetic fields.

We find that the distribution of the stimulated emitted phonons are similar to Lorentzian shape, so $H$ can be an additional degree of freedom to control phonon laser. And that phenomenon similar to a switch of a phonon laser can be obtained by adjusting $H$ without changing other parameters. Especially, the width of the windows almost does not change with the loss of magnon mode ${\kappa }_{m}$. Moreover, it can be seen that the number of phonons increase with decreasing ${\kappa }_{m}$.

**Fig. 5:**
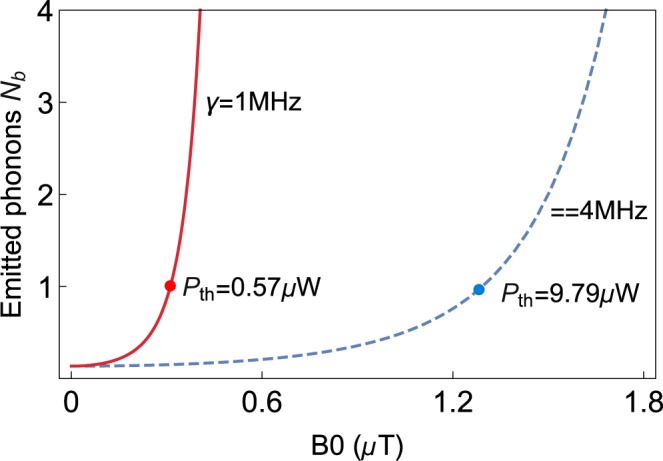
The stimulated emitted phonon number ${n}_{b}$ versus the drive magnetic field ${B}_{0}$ under $\gamma =1\ MHz$ (red solid line, ${P}_{th}=0.57\ \mu$W) and $\gamma =4\ MHz$ (blue dashed line, ${P}_{th}=9.79\ \mu$W). The other parameter we used is ${\Delta }_{m}/2\pi =8\ MHz.$

A plot of the phonon number ${N}_{b}$ versus the drive magnetic field ${B}_{0}$ is shown in Fig. 5, the stimulated emitted phonons are enhanced by input drive magnetic field. Here, threshold condition of phonon laser is ${N}_{b}=1$. Physically, the increase of drive magnetic field ${B}_{0}$ leads to the enhancement of magnon mode. Thus the magnetostrictive effect is also strengthened effectively^21^, and the number of phonons is eventually increased.

In addition, we find the loss of supermode has significant impact on the threshold power ${P}_{th}$. For $\gamma =1\ MHz$ and $\gamma =4\ MHz$, we have ${P}_{th} \sim 0.57\ \mu$W and ${P}_{th} \sim 9.79\ \mu$W, respectively. It is worth mentioning that ${P}_{th}$ is calculated in the range of parameters that can be achieved by experiments, and it is not much different from the ${P}_{th}$ obtained in the cavity optomechanics system, and the threshold power in cavity optomechanical system is generally about $7\ \mu$W in^38–41,56^.

## Discussion

In summary, we have studied theoretically phonon laser in a cavity magnomechanical system, the interaction between magnon mode and mechanical mode can be achieved by magnetostrictive force (radiation pressure like). Our results have shown that a window with a large number of phonons can be obtained by adjusting the intensity of external magnetic field $H$. And the width of the window is about $0.5\ mT$. Compared with phonon laser in optomechanical systems, our scheme brings a new degree of freedom of manipulation.

We first use the way of resonantly driving the YIG sphere, which can be realized by a microwave source. A threshold condition of drive magnetic field intensity for phonon laser is given. Then, we find the system can reach the threshold power and produce phonon laser by increasing the intensity of drive magnetic field ${B}_{0}$. With the experimentally feasible parameters, threshold power ${P}_{th}$ in our system is close to the threshold power of phonon laser in optomechanical systems which are mature in theory and experiments. Finally, with the advance in quantum theory and technology, we hope that the phonon laser in cavity magnomechanical systems will be accessible.

## Methods

### The mechanical gain of system

A phonon laser can be obtained theoretically with two coupled whispering-gallery-mode microtoroid resonators via inversion of the two supermodes^38,57^, where the threshold power of phonon laser is ${P}_{th} \sim 7\ \mu$W. Similarly, our system has two supermodes corresponding to the ground and excited states, respectively, the phonon mode can realize energy level transition. Using magnetic pumping of the upper level, the phenomenon of stimulated emission of phonon appears, then coherent phonon lasing can be realized. Therefore, we introduce supermode operators ${\Re }_{\pm }=(a\pm {m}^{\dagger })/\sqrt{2}$ to rewrite the Hamiltonian ${H}_{0}$ and ${H}_{d}$ of the system, i.e.,

5 $$\begin{array}{lll}{H}_{0,sm} & = & {\omega }_{+}{\Re }_{+}^{\dagger }{\Re }_{+}+{\omega }_{-}{\Re }_{-}^{\dagger }{\Re }_{-}+{\omega }_{b}{b}^{\dagger }b,\\ {H}_{d,sm} & = & i/\sqrt{2}[{\varepsilon }_{d}({\Re }_{+}^{\dagger }+{\Re }_{-}^{\dagger })-{\varepsilon }_{d}^{* }({\Re }_{+}+{\Re }_{-})],\end{array}$$

where the supermode frequencies ${\omega }_{\pm }=-\frac{\Delta }{2}\pm {g}_{ma}$. ${H}_{int}$ in Eq. (1) can be tranformed to

6 $$\begin{array}{ccc}{H}_{int} & = & -\frac{{g}_{mb}}{2}({n}_{+}+{n}_{-}+{\Re }_{+}^{\dagger }{\Re }_{-}+{\Re }_{+}{\Re }_{-}^{\dagger })(b+{b}^{\dagger })\\  &  & +{g}_{ma}({n}_{+}-{n}_{-}),\end{array}$$

with ${n}_{+}={\Re }_{+}^{\dagger }{\Re }_{+}$ and ${n}_{-}={\Re }_{-}^{\dagger }{\Re }_{-}$. Applying the rotating-wave approximation, ${H}_{int}$ is rewritten as

7 $${H}_{int,sm}=-\frac{{g}_{mb}}{2}({p}^{\dagger }b+p{b}^{\dagger }),$$

where $p={\Re }_{+}{\Re }_{-}^{\dagger }$ is ladder operator. Equation (7) represents the absorption and emission of phonons. In general, the introduction of supermode operators ${\Re }_{\pm }$ means that the magnon mode and the optical mode have the same resonant frequency. After changing the Hamiltonian into the supermode picture, the equations of motion read

8 $$\begin{array}{lll}{\dot{\Re }}_{+} & = & -(i{\omega }_{+}+\gamma ){\Re }_{+}+\frac{i}{2}{g}_{mb}b{\Re }_{-}+\frac{{\varepsilon }_{d}}{\sqrt{2}},\\ {\dot{\Re }}_{-} & = & -(i{\omega }_{-}+\gamma ){\Re }_{-}+\frac{i}{2}{g}_{mb}{b}^{\dagger }{\Re }_{+}+\frac{{\varepsilon }_{d}}{\sqrt{2}},\\ \dot{b} & = & -(i{\omega }_{b}+{\gamma }_{b})b+\frac{i}{2}{g}_{mb}p,\\ \dot{p} & = & -2(\gamma +i{g}_{ma})p-\frac{i}{2}{g}_{mb}b\Delta n+\frac{1}{\sqrt{2}}({\varepsilon }_{d}{\Re }_{-}^{\dagger }+{\varepsilon }_{d}^{* }{\Re }_{+}),\end{array}$$

where $\gamma =({\kappa }_{a}+{\kappa }_{m})/2$, and $\Delta n={n}_{+}-{n}_{-}$ is an inversion operator. With the steady state condition of Eq. (8), the zero-order steady states of the system are given by

9 $$\begin{array}{lll}{\Re }_{+,s} & = & \frac{\sqrt{2}{\varepsilon }_{d}[2r+i(2{\omega }_{-}+b{g}_{mb})]}{4({\gamma }^{2}+{g}_{ma}^{2})-{\Delta }^{2}+{g}_{mb}^{2}{b}^{\dagger }b-4i\gamma \Delta },\\ {\Re }_{-,s} & = & \frac{\sqrt{2}{\varepsilon }_{d}[2r+i(2{\omega }_{+}+{b}^{\dagger }{g}_{mb})]}{4({\gamma }^{2}+{g}_{ma}^{2})-{\Delta }^{2}+{g}_{mb}^{2}{b}^{\dagger }b-4i\gamma \Delta },\\ p & = & \frac{\sqrt{2}(({\varepsilon }_{d}{ {\hat{a}} }_{-}^{\dagger }+{\varepsilon }_{d}^{* }{a}_{+}))-i{g}_{mb}b\Delta n}{4\gamma +i(4{g}_{ma}-2{\omega }_{b})},\end{array}$$

where $\Delta ={\Delta }_{m}+{\Delta }_{a}$, then Eq. (9) is substituted into Eq. (8), the result about $b$ can be obtained

10 $$\dot{b}={\Delta }_{b}b+\chi ,$$

with

11 $$\begin{array}{lll}{\Delta }_{b} & = & -i{\omega }_{b}^{{}^{^{\prime} }}-G-{\gamma }_{b},\\ G & = & {g}_{mb}^{2}\gamma [\frac{\Delta n}{8{\gamma }^{2}+2{\eta }^{2}}+\beta ],\\ \beta  & \simeq  & \frac{{\left|{\varepsilon }_{d}\right|}^{2}\eta \Delta }{4({\gamma }^{2}+{g}_{ma}^{2}-\frac{{\Delta }^{2}}{4}+{\Delta }^{2}{\gamma }^{2})\left(\eta +4{\gamma }^{2}\right)},\end{array}$$

where $\eta =2{g}_{ma}-{\omega }_{b}$, and the approximation is due to the parameters we have chosen $({g}_{mb}\ \ll \ \Delta )$. Then the inversion operator can be express as

12 $$\Delta n\simeq \frac{2{g}_{ma}{\left|{\varepsilon }_{d}\right|}^{2}}{{({\gamma }^{2}+{g}_{ma}^{2}-\frac{{\Delta }^{2}}{4})}^{2}+{\gamma }^{2}{\Delta }^{2}}.$$

Because this paper mainly studies the phonon laser generated by the system, we are only interested in mechanical gain $G$, which indicates the mechanical gain of the system. Therefore, only the specific expression of $G$ is obtained. The non-negative $G$ leads to the decrease of ${\gamma }_{eff}$, where ${\gamma }_{eff}={\gamma }_{b}-G$. That makes the instability of the mechanical oscillator at ${\gamma }_{eff} < 0$. Here, ${\gamma }_{eff}$ is effective damping rate of the mechanical mode. This problem has been analyzed and discussed in^38,40,58^ from both theoretical and experimental perspectives.

### The phonon number of system

The stimulated emitted phonon number can be calculated^39,40^, i.e.,

13 $${N}_{b}=\exp [2(G-{\gamma }_{b})/{\gamma }_{b}],$$

then from the above expression, the threshold condition of phonon laser is given (the threshold condition for phonon lasing ${N}_{b}=1$). When ${N}_{b}=1$, we have

14 $${B}_{0,th}=\frac{8\sqrt{\frac{2}{5}{\gamma }_{b}\Gamma }}{{g}_{mb}\sqrt{{g}_{ma}M\gamma \Delta }},$$

where $\Gamma ={g}_{ma}^{4}+2{g}_{ma}^{2}({\gamma }^{2}-\frac{{\Delta }^{2}}{4})+{({\gamma }^{2}+\frac{{\Delta }^{2}}{4})}^{2}$. ${B}_{0,th}$ is the drive magnetic field required to achieve the threshold condition of the phonon laser in our system. Finally, according to the expression given earlier $P=({B}_{0}^{2}/2{\mu }_{0})Ac$, the threshold power is defined as

15 $${P}_{th}=\frac{64}{5}\frac{Ac{\gamma }_{b}\Gamma }{{g}_{mb}^{2}{g}_{ma}M\gamma {\mu }_{0}\Delta }.$$
